# Seomae mugwort and jaceosidin attenuate osteoarthritic cartilage damage by blocking IκB degradation in mice

**DOI:** 10.1111/jcmm.15471

**Published:** 2020-06-11

**Authors:** Hyemi Lee, Dain Jang, Jimin Jeon, Chanmi Cho, Sangil Choi, Seong Jae Han, Eunjeong Oh, Jiho Nam, Chan Hum Park, Yu Su Shin, Seung Pil Yun, Siyoung Yang, Li‐Jung Kang

**Affiliations:** ^1^ Department of Biomedical Sciences Ajou University Graduate School of Medicine Suwon Korea; ^2^ Department of Pharmacology Ajou University School of Medicine Suwon Korea; ^3^ CIRNO Sungkyunkwan University Suwon Korea; ^4^ Department of Medicinal Crop Research National Institute of Horticultural and Herbal Science Rural Development Administration Eumseong Korea; ^5^ Department of Pharmacology and Convergence Medical Science Institute of Health Science, School of Medicine, Gyeongsang National University Jinju Korea

**Keywords:** cartilage destruction, IκB, jaceosidin, matrix metalloproteinase, nuclear factor‐kappa B, osteoarthritis, Seomae mugwort

## Abstract

Seomae mugwort, a Korean native variety of *Artemisia argyi*, exhibits physiological effects against various diseases. However, its effects on osteoarthritis (OA) are unclear. In this study, a Seomae mugwort extract prevented cartilage destruction in an OA mouse model. In vitro and ex vivo analyses revealed that the extract suppressed MMP3, MMP13, ADAMTS4 and ADAMTS5 expression induced by IL‐1β, IL‐6 and TNF‐α and inhibited the loss of extracellular sulphated proteoglycans. In vivo analysis revealed that oral administration of the extract suppressed DMM‐induced cartilage destruction. We identified jaceosidin in Seomae mugwort and showed that this compound decreased MMP3, MMP13, ADAMTS4 and ADAMTS5 expression levels, similar to the action of the Seomae mugwort extract in cultured chondrocytes. Interestingly, jaceosidin and eupatilin combined had similar effects to Seomae mugwort in the DMM‐induced OA model. Induction of IκB degradation by IL‐1β was blocked by the extract and jaceosidin, whereas JNK phosphorylation was only suppressed by the extract. These results suggest that the Seomae mugwort extract and jaceosidin can attenuate cartilage destruction by suppressing MMPs, ADAMTS4/5 and the nuclear factor‐κB signalling pathway by blocking IκB degradation. Thus, the findings support the potential application of Seomae mugwort, and particularly jaceosidin, as natural therapeutics for OA.

## 1 INTRODUCTION

*Artemisia argyi* is a species of mugwort and a natural herb used in food, tea and traditional medicine in East Asia. It provides health benefits via its antioxidant, anti‐inflammatory and gastroprotective activities.^1^, ^2^, ^3^ *A argyi* contains jaceosidin and eupatilin.^4^ Eupatilin is used to prevent and treat bone diseases, cardiovascular diseases, glioma and obesity.^5^, ^6^, ^7^, ^8^ Jaceosidin reduces the oxidation of low‐density lipoprotein, which is related to the inflammatory processes of atherosclerosis.^9^ Seomae mugwort is a Korean native variety of *A argyi* that contains various compounds, such as volatile chemicals, polyunsaturated fatty acids, phenolic compounds, vitamin C and essential amino acids.^10^ However, the effect of Seomae mugwort on osteoarthritis (OA) remains largely unknown.

Osteoarthritis is a common degenerative joint disease. It is a complex disease with pathological features of cartilage destruction, joint stiffness and pain.^11^ OA is difficult to diagnose,^12^ warranting a focus on prophylactic approaches. Few natural substances or foods with prophylactic effects for OA have been identified. Glucosamine, a sugar‐containing protein found in bone, shellfish and fungi, helps build connective tissue and is used in alternative medicine. Although the protective benefits of glucosamine for OA have been reported,^13^, ^14^ the Food and Drug Administration has not approved its use in OA, given the lack of evidence of efficacy.^15^ According to a 2010 meta‐analysis, glucosamine was no better than the placebo in OA protection or prevention.^16^ In addition, side effects that include allergic reactions, diarrhoea, nausea and heartburn have been described.^17^

The extracellular matrix (ECM) is a component of the cartilage that contains chondrocytes. The main ECM components of cartilage are collagens and proteoglycan, which are synthesized by chondrocytes and degraded by enzymes secreted from chondrocytes.^18^ The synthesis and degradation of ECM maintains the balance of cartilage under normal conditions. However, abnormal chondrocytes subjected to specific types of stimuli secrete large amounts of degrading enzymes, such as matrix metalloproteinases (MMPs), a disintegrin and metalloproteinase with thrombospondin motifs (ADAMTSs), which are the primary enzymes that degrade the ECM and promote cartilage destruction.^19^ In addition, pro‐inflammatory cytokines, including interleukin (IL)‐1β, induce abnormal conditions for chondrocytes, which, in turn, induces the up‐regulation of catabolic factors.^20^

IL‐1β, IL‐6 and TNF‐α play a crucial role in joint destruction by inducing MMPs and ADAMTSs, being related to the pathogenesis of OA.^21^ Treatment with IL‐1β in both human normal and OA chondrocytes reportedly up‐regulates MMP3, MMP13, ADAMTS4 and ADAMTS5.^22^, ^23^ Accordingly, IL‐1β, IL‐6 and TNF‐α have been used to induce OA mimetic conditions and up‐regulate MMP3, MMP13, ADAMTS4 and ADAMTS5, which are pivotal proteinases related to OA, as well as to degrade collagens and aggrecans.^19^ IL‐1β, IL‐6 and TNF‐α also activate nuclear factor‐kappa B (NF‐κB) signalling in chondrocytes, which promotes OA pathogenesis.^24^

As a transcription factor, NF‐κB has a key regulatory role in the expression of genes, including those encoding cytokines, chemokines, growth factors and various enzymes.^25^, ^26^ In OA, the increased expression of ECM‐degrading enzymes, including MMPs and ADAMTSs, is affected by activated NF‐κB signalling, where NF‐κB is separated from the IκB protein. This protein inactivates NF‐κB, and the separated IκB is degraded.^27^ Although drugs such as aspirin and sulfasalazine regulate NF‐κB signalling,^28^ they have gastrointestinal side effects, such as an upset stomach and vomiting.^29^

Considering this background, the present study was performed to investigate the prophylactic effect of this natural extract on OA pathogenesis.

## 2 MATERIALS AND METHODS

### 2.1 Reagents and treatment

We obtained lyophilized Seomae mugwort extract from Namhae Seomae mugwort Agricultural Association Corporation. Jaceosidin and eupatilin were purchased from Cayman Chemical. IL‐1β, IL‐6 and TNF‐α recombinant proteins were purchased from GenScript. Lyophilized Seomae mugwort was dissolved in dimethyl sulfoxide (DMSO), and the recombinant proteins were dissolved in sterilized water. Mouse articular chondrocytes were treated with IL‐1β (1 ng/mL), IL‐6 (50 ng/mL) and TNF‐α (50 ng/mL), and co‐treated with Seomae mugwort at different concentrations (10, 50 and 100 μg/mL) for 24 hours before harvest. After the chondrocytes were treated with IL‐1β (1 ng/mL), IL‐6 (50 ng/mL) and TNF‐α (50 ng/mL), for 12 hours, not for 24 hours, they were incubated in chondrocyte medium containing jaceosidin at different concentrations (10, 20, 40 and 80 μmol/L).

### 2.2 Primary culture of mouse chondrocytes

Epiphyseal cartilage was isolated from the femoral heads, femoral condyles and tibial plateaus of 5‐day‐old ICR mice (DBL). The protocols were approved by the Animal Care and Use Committee of the University of Ajou. Cartilage tissues (1.46 mm) were digested using 0.2% collagenase type II and chondrocytes were cultured in DMEM (Gibco‐BRL) as described previously.^30^ In each in vitro experiment, four replicates were performed.

### 2.3 Cell viability assay

Chondrocytes were extracted from cartilage explants obtained from the knee joints of 5‐day‐old mice. The cells were seeded into a 96‐well dish (1.5 × 10^4^ cells/well). After 24 hours, we evaluated the cell viability using a lactate dehydrogenase (LDH) assay. As described previously,^31^ the experiments were performed using an LDH colorimetric assay kit (BioVision, Inc). For normalization, we used untreated samples (100% viability) and Triton X‐100‐treated samples (0% viability). We examined the supernatants of chondrocytes that were incubated with various concentrations of Seomae mugwort and jaceosidin for 12 and 24 hours. The per cent viability was calculated using the following formula: 100 − (sample LDH − negative control)/(max LDH − negative control) × 100. Each signal was detected using a SYNERGY H1 microplate reader (Biotek) at 495 nm.

### 2.4 RT‐PCR and qRT‐PCR

We used TRIzol (Molecular Research Center, Inc) to isolate total RNA from the articular chondrocytes as described previously.^32^ cDNA was generated by reverse transcription with total RNA, and the cDNA was amplified by PCR (Intron Biotechnology). The primers used for PCR were as follows: mouse MMP3, 5′‐TCCTGATGTTGGTGGCTTCAG‐3′ and 5′‐TGTCTTGGCAAATCCGGTGTA‐3′; mouse MMP13, 5′‐TGATGGACCTTCTGGTCTTCTGG‐3′ and 5′‐CATCCACATGGTTGGGAAGTTCT‐3′; mouse ADAMTS4, 5′‐TCCTGATGTTGGTGGCTTCAG‐3′ and 5′‐TGTCTTGGCAAATCCGGTGTA‐3′; mouse ADAMTS5, 5′‐TCCTGATGTTGGTGGCTTCAG‐3′ and 5′‐TGTCTTGGCAAATCCGGTGTA‐3′; and mouse glyceraldehyde 3‐phosphate dehydrogenase (GAPDH), 5′‐TCACTGCCACCCAGAAGAC‐3′ and 5′‐TGTAGGCCATGAGGTCCAC‐3′. qRT‐PCR using SYBR premix ExTaq (TaKaRa Bio) was performed to quantify the transcript levels of the genes. The transcript levels of each gene were normalized to that of GAPDH.

### 2.5 Protein isolation and Western blotting

We extracted total proteins using lysis buffer (150 mmol/L NaCl, 1% NP‐40, 50 mmol/L Tris, 0.2% sodium dodecyl sulphate and 5 mmol/L NaF) mixed with a protease inhibitor and phosphatase inhibitor (Roche) as described previously.^33^ MMP3 and MMP13 secreted into the culture medium were detected using antibodies against MMP3 (ab52915; Abcam) and MMP13 (ab51072; Abcam) after performing trichloroacetic acid precipitation with conditioned medium. The levels of IκB (#9242S; Cell Signaling Technology (CST)), P38 (#9212; CST), pP38 (#9215S; CST), c‐Jun N‐terminal kinase (JNK) (#9252S; CST), phosphorylated (p)JNK (#9251S; CST) and p‐extracellular signal‐regulated kinase (pERK) (#9101S; CST) were detected in the cell lysates. Protein levels were determined using an anti‐ERK1/2 antibody (610408; BD Biosciences). We quantified the relative intensities of the bands by densitometric analysis (AlphaEase FC 4.0; Alpha Innotech).

### 2.6 Culture of cartilage explants and Alcian blue staining

Cartilage explants (1.46 mm) from mouse knee joints of 5‐day‐old ICR mice (DBL) were cultured in DMEM (Gibco‐BRL) with or without IL‐1β. The Seomae mugwort extract was added at 10, 50 and 100 μg/mL, and the amended medium was changed every 24 hours. After 72 hours, the cartilage explants were fixed with 4% paraformaldehyde, infused with paraffin and embedded in paraffin blocks. Blocks were sectioned at a thickness of 5 μm. Alcian blue staining of the sections was performed to evaluate the accumulation of sulphate proteoglycans as previously described.^30^ In each ex vivo experiment, four replicates were performed.

### 2.7 DMM‐induced OA mouse model and oral administration of the test extract

Animal experiments were approved by the Animal Care and Use Committee of the University of Ajou. We performed destabilization of the medial meniscus (DMM) surgery in 10‐week‐old male C57BL/6 mice according to a previously described protocol.^34^ Experimental OA mice were orally administered Seomae mugwort extract, jaceosidin, eupatilin and the jaceosidin/eupatilin combination dissolved in phosphate‐buffered saline (PBS) or PBS alone (controls) on every other day for 10 weeks. The mice were sacrificed after 10 weeks for histological analysis.

### 2.8 Analysis of ethanol fraction from Seomae mugwort

The water extract of Seomae mugwort was dissolved in 10 mL of 50% methanol with multiple vortexing, followed by filtration through a Dismic‐13 JP membrane filter (Advantec Toyo; pore diameter: 0.2 μm). We injected 10 μL of the sample into a reverse‐phase high‐performance liquid chromatography (HPLC) system using an Agilent ZORBAX Eclipse Plus C18 column (4.6 × 250 mm, 5 μm), with a column temperature of 35°C; mobile phase: A = water, B = acetonitrile. The gradient conditions were as follows: 0 minute, 0% B; 4 minutes, 0% B; 10 minutes, 4% B; 20 minutes, 10% B; 30 minutes, 20% B; 40 minute, 40% B; and 70 minutes, 100% B. The flow rate was 0.8 mL/min. The ultraviolet (UV) absorbance was monitored at 210 nm using a model 1200 series diode array detector (Agilent Technologies). The peak was assigned by carrying out a co‐injection test with an authentic sample and comparing this with the UV spectral data. The measurement was repeated thrice for each sample. Representative HPLC results are provided in Figure 3A.

### 2.9 Safranin O staining and immunohistochemistry

Cartilage destruction was evaluated as previously reported.^35^ Briefly, cartilage was fixed in 4% paraformaldehyde, followed by decalcification with 0.5 mol/L EDTA (pH 8.0) for 2 weeks and embedding in paraffin. The paraffin blocks were serially sectioned at a thickness of 5 μm at 40‐μm intervals. For staining, the deparaffinized sections were immersed in xylene and hydrated with graded ethanol. Sections of knee joints were imaged using a high‐resolution micro‐computed tomography specimen scanner (VivaCT 80; Scanto Medical AG) at the Chuncheon Center of Korea Basic Science Institute. Finally, safranin O staining was performed to evaluate cartilage destruction. The results were scored using the Osteoarthritis Research Society International (OARSI), osteophyte maturity grading system and subchondral bone plate thickness. As previously described,^32^ immunohistochemistry was performed in mouse knee joint section using IκB (#9242S; CST) antibodies.

### 2.10 Aggrecanase activity assay

The supernatants of mouse primary chondrocyte cultures treated with Seomae mugwort or jaceosidin and co‐treated with IL‐1β were used to assay the ADAMTS activity. A commercially available aggrecanase activity assay kit (Abnova) was used according to the manufacturer's protocol. Briefly, the enzyme activity of the test sample was evaluated by capacity digesting a recombinant fragment of aggrecan. The aggrecan digested by ADAMTS revealed a ARGSVIL‐peptide through proteolytic cleavage, which was quantified by ELISA using specific antibodies.

### 2.11 Statistical analysis

Data are shown as the mean ± SEM, and one‐way analysis of variance (ANOVA) along with Bonferroni post hoc tests was used to evaluate the significance of data. Significance was considered at the .05 level of probability (*P* < .05). Statistical analysis was performed using GraphPad Prism 5 software (GraphPad).

## 3 RESULTS

### 3.1 Seomae mugwort does not affect the viability of cultured mouse chondrocytes

Before studying the effect of Seomae mugwort, we examined the cytotoxicity of the Seomae mugwort extract. Lyophilized Seomae mugwort was dissolved in DMSO at 100 mg/mL and used to treat chondrocytes at concentrations of 10, 50 and 100 μg/mL for 12 and 24 hours (Figure S1A). As the control, an equal amount of DMSO was added to the chondrocytes. At both 12 and 24 hours, cell viability did not differ from the control for any of the extract concentrations. Based on these results, the Seomae mugwort extract was used at experimental concentrations of 10, 50 and 100 μg/mL for 24 hours.

### 3.2 Seomae mugwort reduces the IL‐1β‐induced expression of MMP3, MMP13, ADAMTS4 and ADAMTS5

**FIGURE 1:**
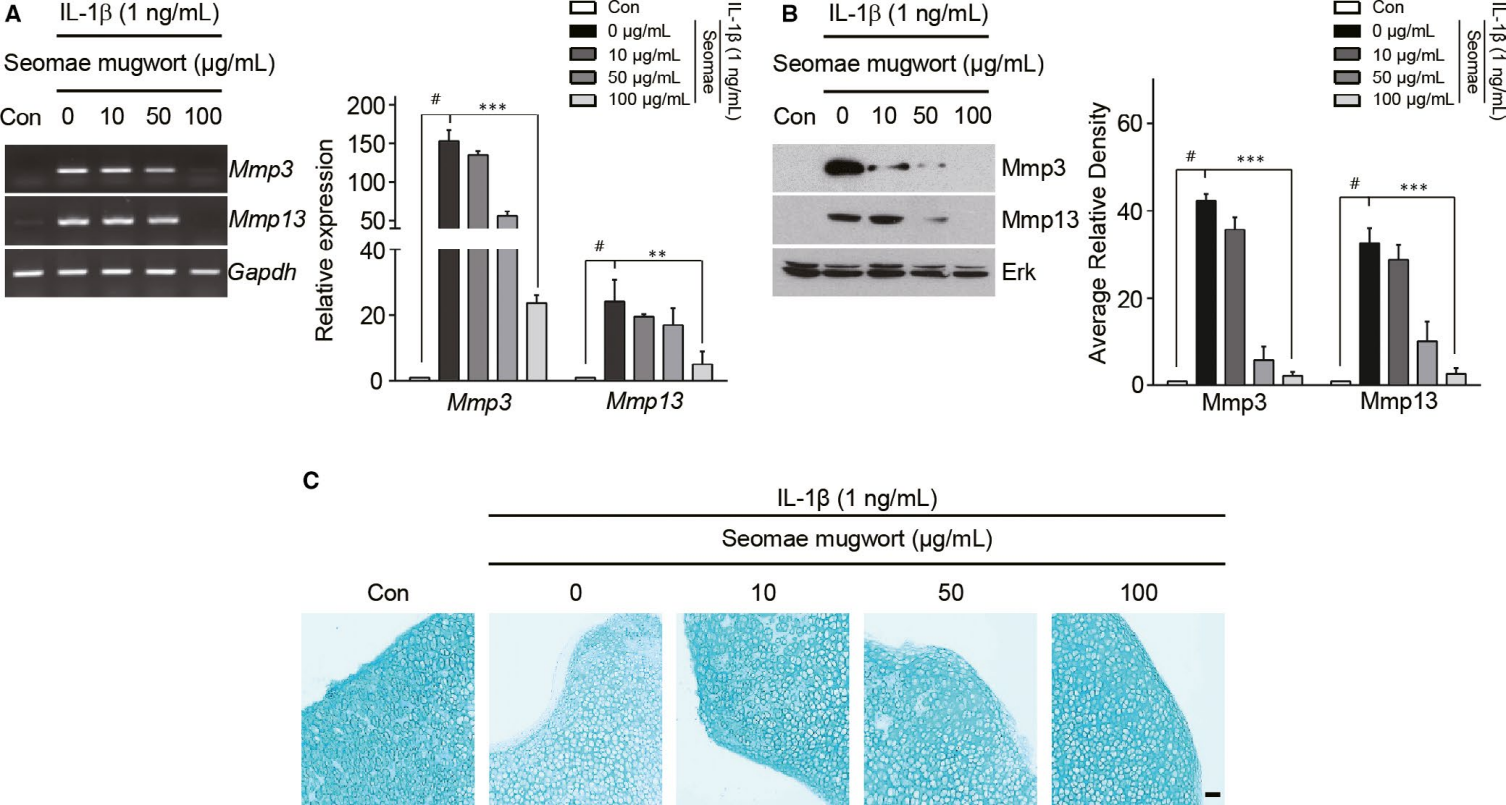
Seomae mugwort extract suppressed the IL‐1β‐induced MMP3 and MMP13 expression in chondrocytes and loss of extracellular sulphate proteoglycans. A and B, Chondrocytes were treated with IL1β (1 ng/mL) and co‐treated with different concentrations of the Seomae mugwort extract for 24 h. MMP3 and MMP13 expression levels were evaluated by PCR (A, left), qRT‐PCR (A, right), Western blotting (B, left) and densitometry (B, right). C, IL‐1β‐stimulated cartilage explants were treated with the Seomae mugwort extract at different doses for 72 h. Alcian blue staining was performed to evaluate the accumulation of sulphate proteoglycans. Scale bar = 100 μm. Values are the mean ± SEM as analysed by one‐way ANOVA with Bonferroni's test (n = 4). Significant differences from the IL‐1β‐treated group value: **P < .01, ***P < .001, #*P* < .05 compared to the control group. Seomae, Seomae mugwort

To determine the effect of Seomae mugwort on the IL‐1β‐induced expression of catabolic factors in vitro, we co‐treated primary mouse chondrocytes with IL‐1β (1 ng/mL) and the Seomae mugwort extract (0, 10, 50 and 100 μg/mL) for 24 hours. Concentration of Seomae mugwort extract for in vitro experiments was based on LDH assays and some studies.^36^, ^37^ PCR revealed that the increased expression levels of MMP3, MMP13, ADAMTS4 and ADAMTS5 were further decreased by the Seomae mugwort extract (Figure 1A and S3). Western blot analysis was performed to assess the changes in the protein levels of these catabolic factors. We extracted proteins from the media to detect MMP3 and MMP13 and confirmed that the Seomae mugwort extract decreased the IL‐1β‐induced secretion of MMP3 and MMP13 (Figure 1B). We also show that Seomae mugwort extract decreased IL‐1β‐induced aggrecanase activity (Figure S3). IL‐6 and TNF‐α are also pro‐inflammatory cytokines that induce the expression of MMP3, MMP13, ADAMTS4 and ADAMTS5 in chondrocytes (Figures S2 and S3). The Seomae mugwort extract also showed a similar effect on the expression of MMP3, MMP13, ADAMTS4 and ADAMTS5 induced by IL‐6 and TNF‐α.

### 3.3 Seomae mugwort protects against the loss of sulphate proteoglycans in IL‐1β‐stimulated cartilage explants

To determine whether Seomae mugwort could protect against the destruction of cartilage, we performed ex vivo experiments using cartilage explants of mouse knee joint. Obtained cartilage explants were cultured for 72 hours with or without IL‐1β and the Seomae mugwort extract. The accumulation of extracellular sulphate proteoglycan was assessed by Alcian blue staining. When the cartilage explants were treated with IL‐1β, the accumulation of sulphate proteoglycan was decreased (Figure 1C). However, co‐treatment with the Seomae mugwort extract and IL‐1β protected against the degradation of sulphated proteoglycan in the cartilage explants. This result corresponded with the results of our in vitro experiments and suggested that Seomae mugwort protects against the loss of extracellular sulphate proteoglycans.

### 3.4 Seomae mugwort suppresses cartilage destruction in a DMM‐induced OA model

**FIGURE 2:**
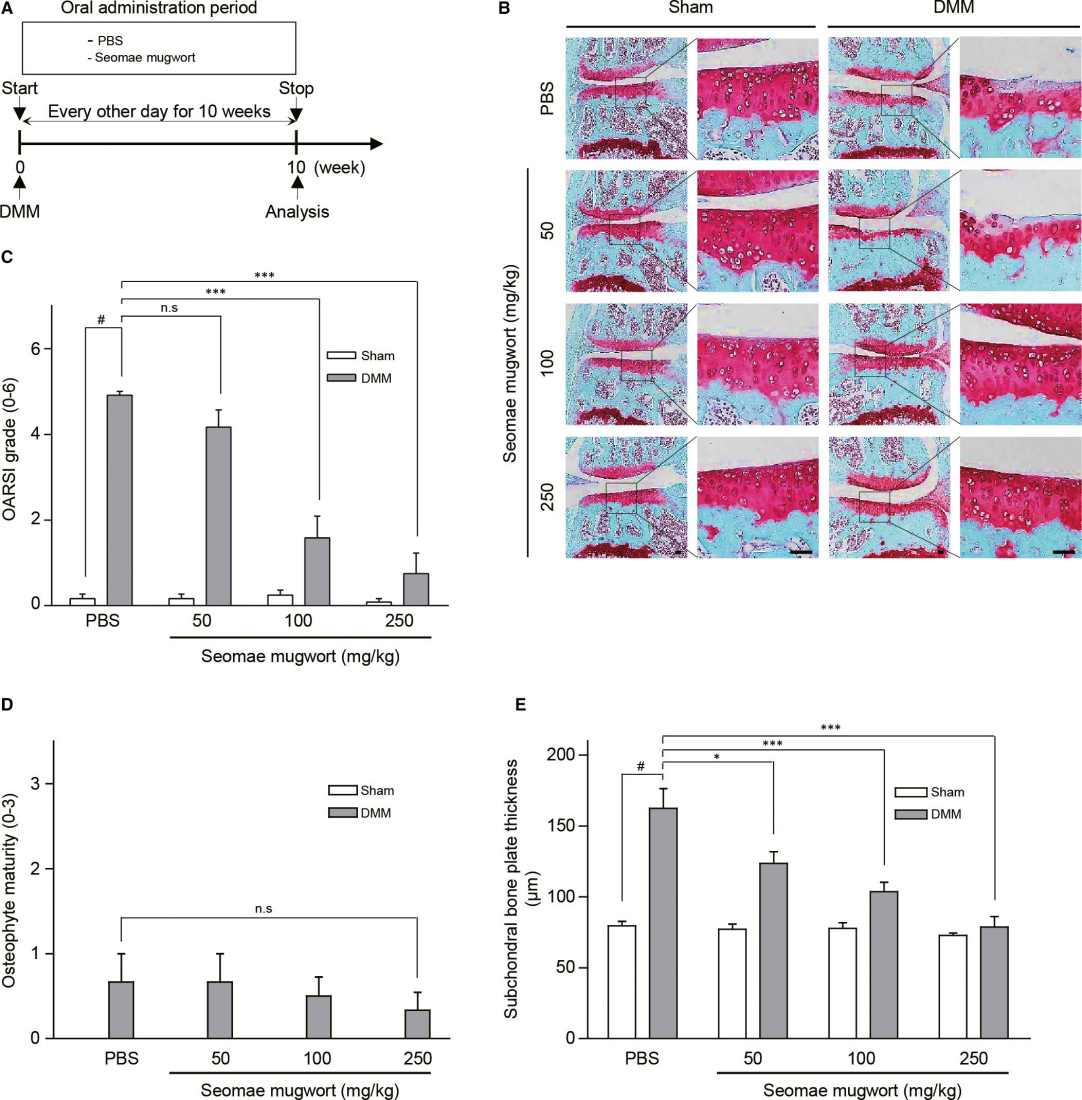
Seomae mugwort extract suppressed the DMM‐induced cartilage degradation. A, Experimental scheme for the analysis of a DMM‐induced OA model. After DMM surgery, the mice were orally administrated the Seomae mugwort extract or PBS every other day for 10 wk. B, Ten‐week‐old C57BL/6 mice were subjected to DMM. Mice were orally administered PBS or the Seomae mugwort extract for 10 weeks after DMM. Cartilage degradation was shown by safranin O staining. Scale bar = 100 μm. C‐E, The severity of OA was evaluated by OARSI scores (C), osteophyte maturity (D) and subchondral bone plate thickness (E) at 10 weeks after DMM. Mice orally administered the Seomae mugwort extract were compared with those administered PBS. Values are the mean ± SEM as analysed by one‐way ANOVA with Bonferroni's test (n = 10 mice per group). Significant differences from the DMM group value: *P < .05, **P < .01, ***P < .001, #*P* < .05 compared to the control group

To determine the effect of the Seomae mugwort extract on OA development in the mouse knee joint, we carried out DMM in mice. Mice were orally administered PBS or the Seomae mugwort extract dissolved in PBS at 50, 100 or 250 mg/kg (Figure 2A). Oral administration of the Seomae mugwort extract significantly protected against DMM‐induced OA compared to the PBS group (Figure 2B). The effect of Seomae mugwort was also evaluated via OARSI scoring (Figure 2C), osteophyte maturity (Figure 2D), and subchondral bone plate thickness (Figure 2E). Seomae mugwort reduced the OARSI score and subchondral bone thickness but did not affect osteophyte maturity. These results are consistent with the in vitro and ex vivo results, suggesting that Seomae mugwort protects the cartilage from destruction by blocking the expression of MMPs and ADAMTSs.

### 3.5 Seomae mugwort is enriched in jaceosidin, and jaceosidin suppresses the IL‐1β‐induced expression of MMP3, MMP13, ADAMTS4 and ADAMTS5

**FIGURE 3:**
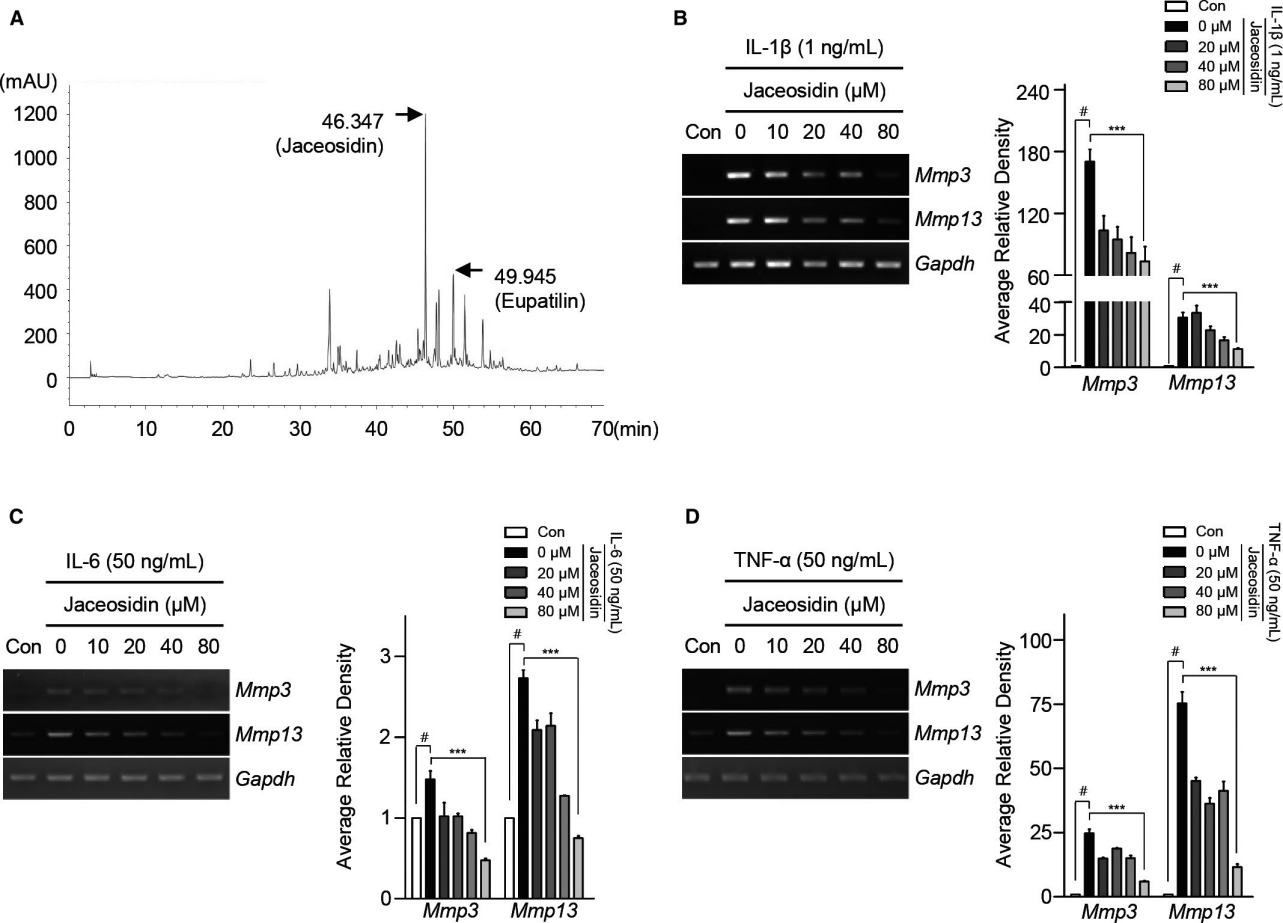
Detection of jaceosidin as a constituent of Seomae mugwort and its suppression of the IL‐1β, IL‐6 and TNF‐α induced expression of MMP3 and MMP13. (A) Results of HPLC analysis of the ethanol extracts of Seomae mugwort. (B‐D) Mouse articular chondrocytes were treated with IL‐1β (1 ng/mL) (B), IL‐6 (50 ng/mL) (C) and TNF‐α (50 ng/mL) (D) and co‐treated with jaceosidin for 12 h. Expression levels of MMP3 and MMP13 were evaluated using PCR (left) and qRT‐PCR (right). Values are the mean ± SEM as analysed by one‐way ANOVA with Bonferroni's test (n = 4). Significant differences from the pro‐inflammatory cytokine‐treated group value: **P < .01, ***P < .001, #*P* < .05 compared to the control group

High‐performance liquid chromatography analysis was performed to identify the natural compounds that are enriched in Seomae mugwort. A previous report indicated that *A argyi* contains jaceosidin and eupatilin.^4^ HPLC of Seomae mugwort revealed the presence of jaceosidin and eupatilin; they were detected at retention times of 46.347 and 49.945 minutes, respectively (Figure 3A). The compounds were quantified by peak area measurement. A calibration curve of standard jaceosidin and eupatilin was generated at the 6.25‐100 μg/mL range. The detector response was linear over this concentration and was described by the calibration equation: area = slope ×concentration + intercept. The correlation coefficient indicated the functional linear relationship between the area under the peak and the concentration. The correlation coefficient (*r* ^2^) of the standard compound exceeded .999 (Table 1). Using peak area and the calibration equation, the concentrations of jaceosidin and eupatilin were calculated (Table 2). HPLC comparative analysis with standard compounds revealed that jaceosidin and eupatilin were the most abundant compounds in the ethanol extract of the aerial portions of Seomae mugwort.

**TABLE 1:** Retention time, calibration equation and correlation coefficients of jaceosidin and eupatilin

Compound	Retention time (min)	Calibration equation ^a^	Correlation coefficient (*r* ^2^)
Jaceosidin (a)	46.347	*y* = 6.4461*x* + 1.2542	.999
Eupatilin (b)	49.945	*y* = 2.2126*x* − 1.0167	.999

^a^ *y* = peak area, *x* = concentration of standards (mg/mL).

**TABLE 2:** Content of jaceosidin (a) and eupatilin (b) in the ethanol extract of the dried aerial portions of Seomae mugwort

Compound	Content (mg/g)	Relative standard deviation (%)
Jaceosidin (a)	130.43 ± 4.21	5.58
Eupatilin (b)	144.66 ± 4.09	4.90

Among the compounds isolated from Seomae mugwort, the effects of eupatilin in OA have been reported, but those of jaceosidin have not. Accordingly, we conducted additional experiments with jaceosidin. An LDH assay revealed that, at the concentrations tested, jaceosidin affected cell viability at 24 hours but not at 12 hours (Figure S1B). To determine the effect of jaceosidin, we treated mouse chondrocytes with IL‐1β (1 ng/mL) and co‐treated them with jaceosidin (0, 20, 40 and 80 μmol/L) for 12 hours. Concentration of jaceosidin for in vitro experiments was based on LDH assays and some studies.^38^, ^39^ PCR revealed that the IL‐1β‐induced MMP3, MMP13, ADAMTS4 and ADAMTS5 expression levels were decreased by jaceosidin in a concentration‐dependent manner. The observations were confirmed using RT‐PCR (Figure 3B‐D and S3). These data suggested that jaceosidin suppressed the IL‐1β‐induced expression of MMP3, MMP13, ADAMTS4, ADAMTS5 and aggrecanase activity.

### 3.6 The jaceosidin and eupatilin components of Seomae mugwort extract suppress cartilage destruction in the DMM‐induced OA model

**FIGURE 4:**
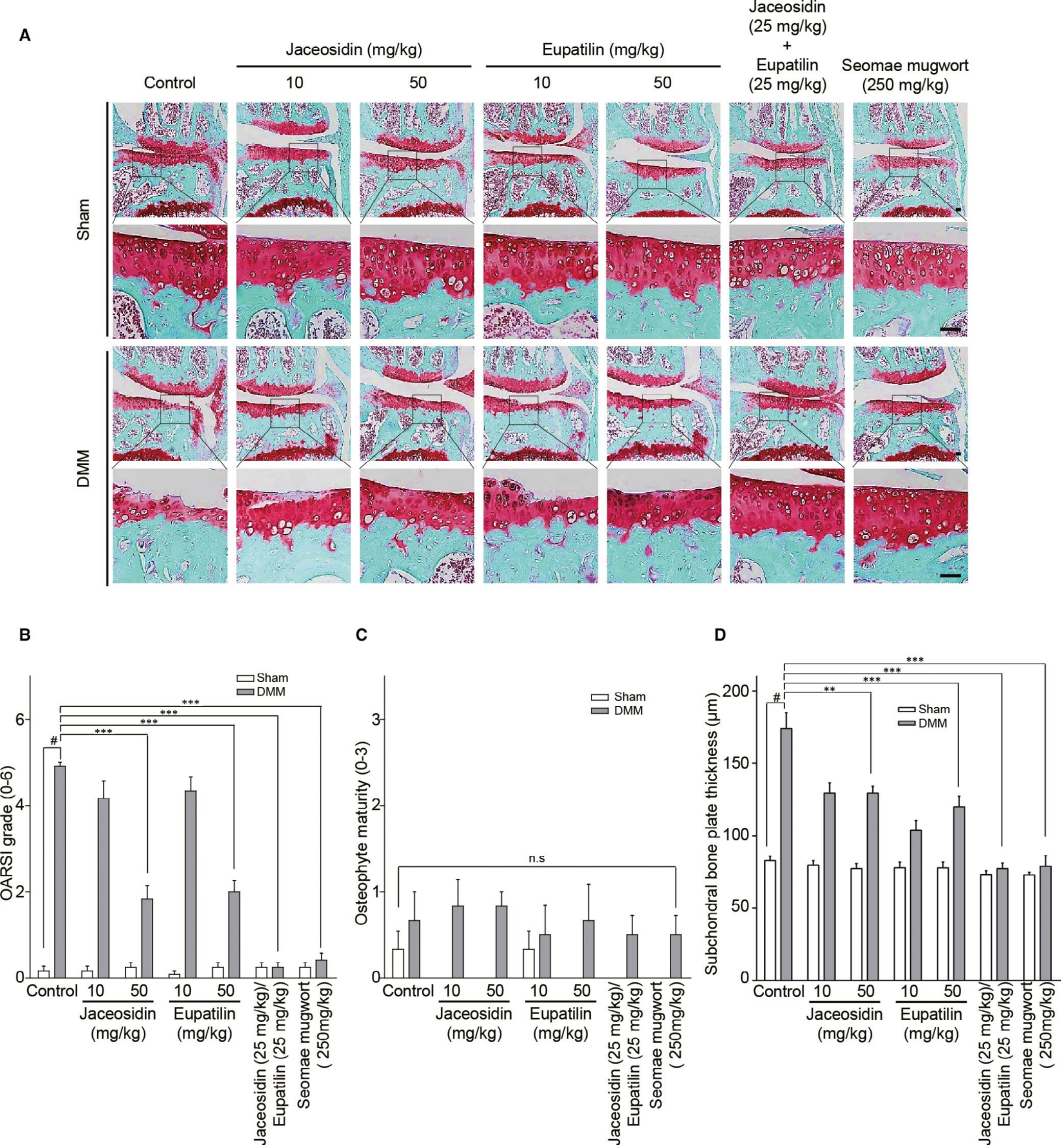
Jaceosidin suppressed the DMM‐induced cartilage degradation. (A‐D) Ten‐week‐old C57BL/6 mice were subjected to DMM. Mice were orally administered PBS, jaceosidin, eupatilin, both jaceosidin and eupatilin, or Seomae mugwort every other day for 10 weeks after DMM. Cartilage degradation was examined by safranin O staining (A). Scale bar = 100 μm. The severity of OA was evaluated by OARSI scores (B), osteophyte maturity (C) and subchondral bone plate thickness (D) at 10 weeks after DMM. Mice orally administered the natural compounds and the Seomae mugwort extract were compared with those administered PBS. Values are the mean ± SEM as analysed by one‐way ANOVA with Bonferroni's test (n = 10 mice per group). Significant differences from the DMM group value: **P < .01, ***P < .001, #*P* < .05 compared to the control group

As in a previous experiment of the Seomae mugwort extract, DMM‐performing mice were orally administrated jaceosidin (50 mg/kg), eupatilin (50 mg/kg), and a mixture of jaceosidin (25 mg/kg) and eupatilin (25 mg/kg) for 10 weeks. Compared with the control group, cartilage destruction was inhibited in the mice administrated jaceosidin. This protection was more effective at a concentration of 50 mg/kg than at 10 mg/kg (Figure 4A). Eupatilin was also effective in repressing cartilage degradation as previously reported. The mixture of jaceosidin and eupatilin was more effective than either compound alone. OARSI grade, osteophyte maturity and subchondral bone plate thickness were also evaluated. Jaceosidin alone and in combination with eupatilin affected the OARSI grade and subchondral bone plate thickness but not osteophyte maturity (Figure 4B‐D).

### 3.7 Jaceosidin and the Seomae mugwort extract suppress the IL‐1β‐induced activation of NF‐κB signalling in mouse chondrocytes

**FIGURE 5:**
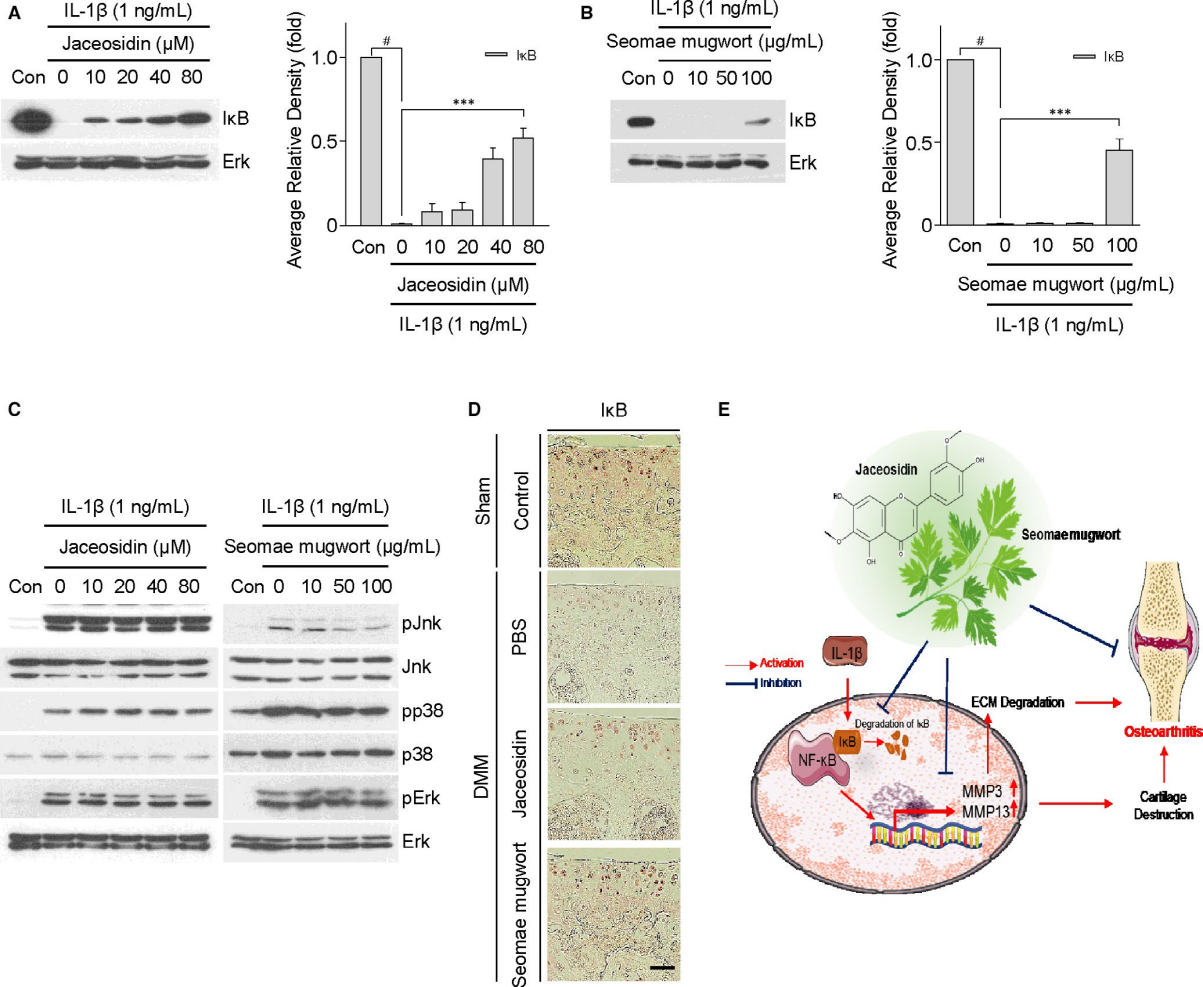
Jaceosidin and the Seomae mugwort extract regulated the expression of catabolic factors via NFκB signalling in mouse articular chondrocytes. A, After treatment with different concentrations of jaceosidin for 12 h, articular chondrocytes were co‐treated with IL‐1β (1 ng/mL) for 10 min. B, After pre‐treatment with different concentrations of the Seomae mugwort extract for 24 h, articular chondrocytes were cotreated with IL‐1β (1 ng/mL) for 10 min. Degradation of IκB was detected by Western blotting (A and B, left) and densitometry analysis (A and B, right). C, Activation of MAPK signalling and NF‐κB was evaluated by Western blotting after jaceosidin (C, left) and Seomae mugwort (C, right). D, Degradation of IκB was detected by immunohistochemistry analysis in oral administration of jaceosidin and Seomae mugwort to DMM‐induced OA mice. Scale bar = 100 μm. Values are the mean ± SEM as analysed by one‐way ANOVA with Bonferroni's test (n = 4). Significant differences from the IL‐1β‐treated group value: ***P < .001, #*P* < .001 compared to the control group. E, Schematic diagram of the signalling pathway leading to NF‐κB inhibition via blocking of IκB degradation by jaceosidin and Seomae mugwort

IL‐1β activates the NF‐κB and MAPK signalling pathways in chondrocytes and is related to OA pathogenesis^24^ (Figure S4). To determine which pathway is related to jaceosidin and Seomae mugwort, we treated primary mouse chondrocytes with or without jaceosidin and the Seomae mugwort extract and co‐treated them with IL‐1β (1 ng/mL) for 10 minutes before cell harvest. Jaceosidin suppressed the degradation of IκB, an NF‐κB subunit that suppresses NF‐κB signalling (Figure 5A). Seomae mugwort also displayed a similar effect (Figure 5B). Jaceosidin did not affect the MAPK pathway, but Seomae mugwort decreased pJNK (Figure 5C). Immunohistochemistry was performed to evaluate the degradation of IκB in knee joints. Jaceosidin and Seomae mugwort suppressed the degradation of IκB (Figure 5D). These results suggested that Seomae mugwort and jaceosidin have an inhibitory effect against OA development by suppressing IL‐1β‐mediated NF‐κB signalling (Figure 5E).

## 4 DISCUSSION

Osteoarthritis is a degenerative disease in which the articular cartilage is degraded, typically resulting in pain.^40^ Prevention and treatment of OA are hindered by difficulties in its early diagnosis.^12^ Novel biological agents that relieve pain have been found, but they do not block OA progression and cause side effects that include headache, upper respiratory tract infection and paresthesia.^41^ Given the difficulties in the diagnosis and treatment of OA, identification of prophylactic substances has been suggested.^12^ The identification of safe and effective therapeutic material for OA is necessary. Natural compounds that are safe and capable of maintaining cartilage health have therefore been given increased focus.

The present study is the first, to our knowledge, to evaluate the effect of the Seomae mugwort extract on OA mimetic conditions. The extract decreased the IL‐1β‐induced expression of MMP3, MMP13, ADAMTS4 and ADAMTS5 and attenuated the NF‐κB signalling pathway. In addition, the Seomae mugwort extract increased the IL‐1β‐induced loss of sulphate proteoglycans in cartilage explants and suppressed the DMM‐induced cartilage destruction.

The effect of Seomae mugwort, a variety of *A argyi*, on OA progression has not been previously addressed in detail. HPLC analysis revealed the presence of large amounts of jaceosidin and eupatilin in the Seomae mugwort extract. Compounds isolated from *A argyi* have antitumor effects,^42^ and liquid‐state fermentation of *A argyi* yields substances that suppress mitochondrial injury and cellular damage by oxidation.^43^

IL‐1β, IL‐6 and TNF‐α have been used to mimic OA because the expression of these cytokines are reportedly increased in OA and modulate various catabolic factors.^44^, ^45^, ^46^, ^47^, ^48^ Chondrocytes exposed to IL‐1β, IL‐6 and TNF‐α release catabolic factors such as MMP3, MMP13, ADAMTS4 and ADAMTS5.^21^, ^49^, ^50^ These factors degrade the ECM, including various collagens and proteoglycans.^19^ MMP3, MMP13, ADAMTS4 and ADAMTS5 are highly expressed in osteoarthritic cartilage compared to normal cartilage, and MMP3 induces the expression of other MMPs.^51^, ^52^, ^53^, ^54^ Thus, MMP3, MMP13, ADAMTS4 and ADAMTS5 are candidate markers of OA. In the present study, the Seomae mugwort extract suppressed the IL‐1β‐, IL‐6‐ and TNF‐α‐induced osteoarthritic process such as up‐regulation of MMP3, MMP13, ADAMTS4 and ADAMTS5 expression.

DMM surgery can induce OA mimetic conditions in vivo, generating a useful model for studying OA pathogenesis that occurs slowly and resembles human OA.^34^ In our DMM model, we found that Seomae mugwort extract protected against DMM‐induced cartilage destruction. Seomae mugwort mainly contains jaceosidin and eupatilin. To determine the effects of these compounds individually, we show that oral administration of jaceosidin (50 mg/kg), eupatilin (50 mg/kg) and jaceosidin (25 mg/kg)/eupatilin (25 mg/kg) combination each prevented osteoarthritic cartilage destruction. However, we found that the OA protective effect of each individual compound was less than that of the jaceosidin/eupatilin combination. Interestingly, the feeding conversion formula is ‘animal dose (mg/kg) × (K_m_ ratio)’.^55^ The doses of jaceosidin (50 mg/kg) and Seomae mugwort extract (250 mg/kg) in mice is similar to the daily dose in humans (17.5 and 43.75 mg/kg, respectively). Furthermore, 10, 50 and 100 μg/mL of Seomae mugwort contained 3043, 6.5215 and 13.043 μg/mL, respectively, of jaceosidin and 1.4466, 7.2233 and 14.455 μg/mL, respectively, of eupatilin (Table 2). Each compound probably corresponds to 10%‐15% of the Seomae mugwort extract. Moreover, previous studies suggest that jaceosidin and eupatilin are present at 10‐80 μmol/L in in vitro^38^, ^39^ and in vivo^56^, ^57^ analyses.

ECM degradation caused by increases in catabolic factors results in cartilage destruction.^33^ ECM is produced and maintained by chondrocytes.^58^ The total amount of proteoglycan fragments in the synovial fluid after knee joint injury is higher than that in the normal joint.^59^ This is because proteoglycans, which protect cartilage against impact‐related injury by binding to water, are typically degraded in the early stages of OA.^60^ In the present study, the Seomae mugwort extract also suppressed the loss of sulphated proteoglycans by simulating IL‐1β. Thus, Seomae mugwort may help to maintain cartilage function.

HPLC revealed the high levels of the eupatilin and jaceosidin in Seomae mugwort. Eupatilin suppresses the TNF‐α‐induced expression of MMPs by reducing NF‐κB signalling in HaCaT human epidermal keratinocytes and prevents skin ageing.^61^ Eupatilin also reportedly suppresses the up‐regulation of pro‐inflammatory cytokines and oxidative damage and protects against cartilage degradation.^57^ NF‐κB signalling is one of the main signalling pathways; it activates and increases the expression levels of catabolic factors.^62^ Jaceosidin regulates angiogenesis by activating the NF‐κB signalling pathway,^63^ but the function of jaceosidin in OA has not yet been studied. We performed in vitro experiments to determine the effect of jaceosidin in OA. Similar to the effects of the Seomae mugwort extract, jaceosidin suppressed the expression of MMPs and ADAMTSs and the reduction of IκB.

IL‐1β also activates the mitogen‐activated protein (MAP) kinase subtypes (extracellular signal‐regulated kinase (ERK), P38 and JNK) in chondrocytes.^64^ c‐JNK phosphorylation of c‐Jun N‐terminal kinase (pJNK) was suppressed by Seomae mugwort, but not by Jaceosidin, in a dose‐dependent manner. Moreover, inhibition of ERK and P38 MAP kinase had no effect on Seomae mugwort and Jaceosidin treatment in chondrocytes. It is possible that eupatilin, a single component of the Seomae mugwort extract, suppresses the JNK pathway.^57^

In conclusion, the collective findings suggest that jaceosidin, one of the compounds isolated from this herb, has an anti‐osteoarthritic effect and suppresses IL‐1β‐induced catabolic factor expression, possibly via blocking IκB degradation. Therefore, the application of the extracts of *A argyi* varieties, such as Seomae mugwort, and jaceosidin should be explored to develop prophylactic and therapeutic agents for OA.

## CONFLICT OF INTEREST

The authors declare no conflict of interest.

## AUTHOR CONTRIBUTION

**Hyemi Lee:** Conceptualization (equal); Formal analysis (lead); Investigation (lead); Validation (lead); Writing‐original draft (lead); Writing‐review & editing (lead). **Dain Jang:** Formal analysis (supporting); Investigation (supporting). **Jimin Jeon:** Conceptualization (supporting); Data curation (supporting); Formal analysis (supporting); Investigation (supporting). **Chanmi Cho:** Conceptualization (supporting); Data curation (supporting); Formal analysis (supporting); Investigation (supporting). **Sangil Choi:** Data curation (supporting); Formal analysis (supporting); Investigation (supporting). **Seong Jae Han:** Conceptualization (supporting); Data curation (supporting); Formal analysis (supporting); Investigation (supporting). **Eunjeong Oh:** Data curation (supporting); Formal analysis (supporting); Investigation (supporting). **Jiho Nam:** Data curation (supporting); Formal analysis (supporting); Investigation (supporting). **Chan Hum Park:** Conceptualization (supporting); Investigation (supporting). **Yu Su Shin:** Conceptualization (supporting); Funding acquisition (equal); Resources (supporting); Supervision (supporting); Writing‐original draft (equal); Writing‐review & editing (equal). **Seung Pil Yun:** Conceptualization (equal); Data curation (equal); Formal analysis (equal); Funding acquisition (supporting); Investigation (supporting); Resources (lead); Writing‐original draft (equal); Writing‐review & editing (equal). **Siyoung Yang:** Conceptualization (lead); Investigation (lead); Project administration (lead); Resources (lead); Writing‐original draft (supporting); Writing‐review & editing (supporting). **Li‐jung Kang:** Conceptualization (equal); Data curation (lead); Formal analysis (equal); Funding acquisition (lead); Investigation (lead); Validation (equal); Writing‐original draft (lead); Writing‐review & editing (lead).

## Supporting information

FigS1‐S4

## Data Availability

Data available on request from the authors.
