# Supplementary material for: Seomae mugwort and jaceosidin attenuate osteoarthritic cartilage damage by blocking IκB degradation in mice
Source: J Cell Mol Med. 2020 Jun 11;24(14):8126–37. doi: 10.1111/jcmm.15471 (PMC7348148; doi:10.1111/jcmm.15471)
Supplement: Supplementary file 1 — FigS1‐S4 [file JCMM-24-8126-s001.pdf]

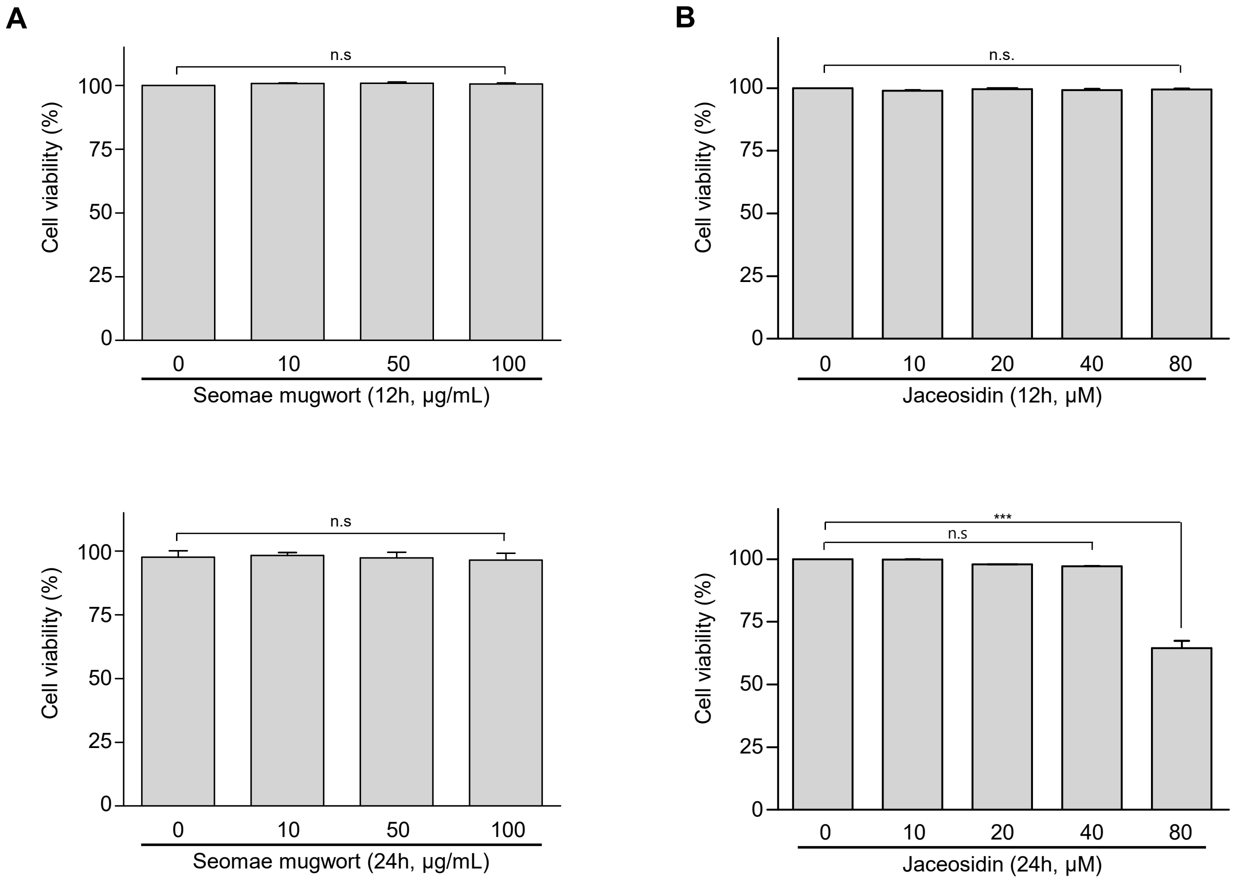

Figure S1. Seomae mugwort extract and jaceosidin did not affect cell viability in mouse articular chondrocytes. (**A**) The Seomae mugwort extract was used to treat articular chondrocytes at concentrations of 0, 10, 50, and 100 μg/mL for 12 h and 24 h and analyzed using the LDH assay. (**B**) Articular chondrocytes were treated with jaceosidin at concentrations of 0, 10, 20, 40, and 80 μM for 12 h and 24 h and analyzed using the LDH assay. Values are the mean ± SEM as analysis by one-way ANOVA with Bonferroni's test (n = 4). Significant differences from the pro-inflammatory cytokine-treated group value: **P < 0.01, ***P < 0.001, #P < 0.05 compared to the control group.

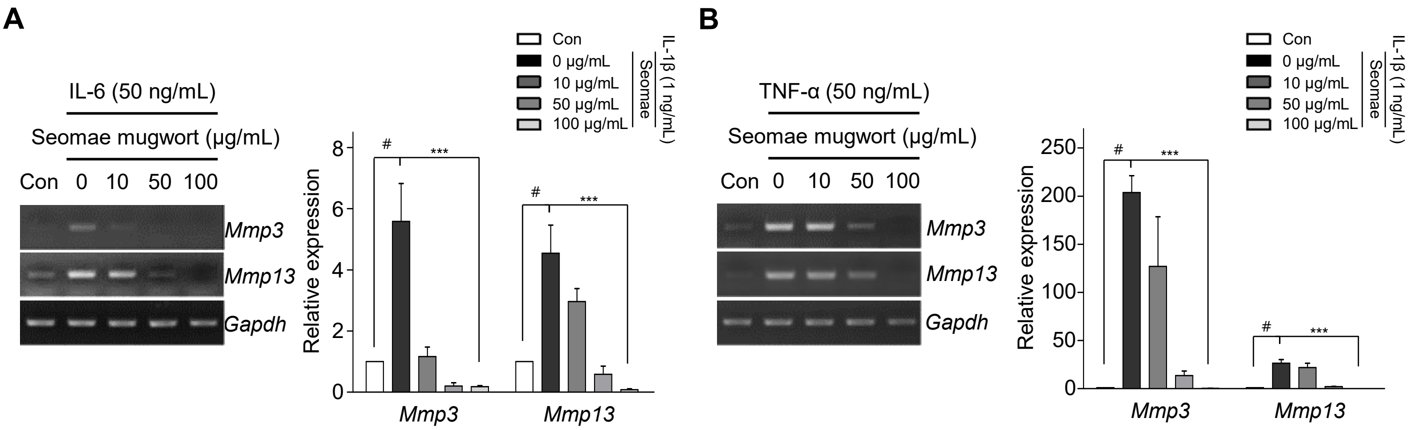

Figure S2. Seomae mugwort extract suppressed the pro-inflammatory cytokine-induced MMP3 and MMP13 expression in chondrocytes. (**A, B**) Chondrocytes were treated with IL-6 (50 ng/mL) (**A**) and TNF-α (50 ng/mL) (**B**) and co-treated with different concentrations of the Seomae mugwort extract for 24 h. MMP3 and MMP13 expression levels were evaluated by PCR (**A**, **B**; left) and qRT-PCR (**A**, **B**; right). Values are the mean ± SEM as analyzed by one-way ANOVA with Bonferroni's test. Significant differences from the pro-inflammatory cytokine-treated group value: **P < 0.01, ***P < 0.001, #P < 0.05 compared to the control group. Seomae, Seomae mugwort.

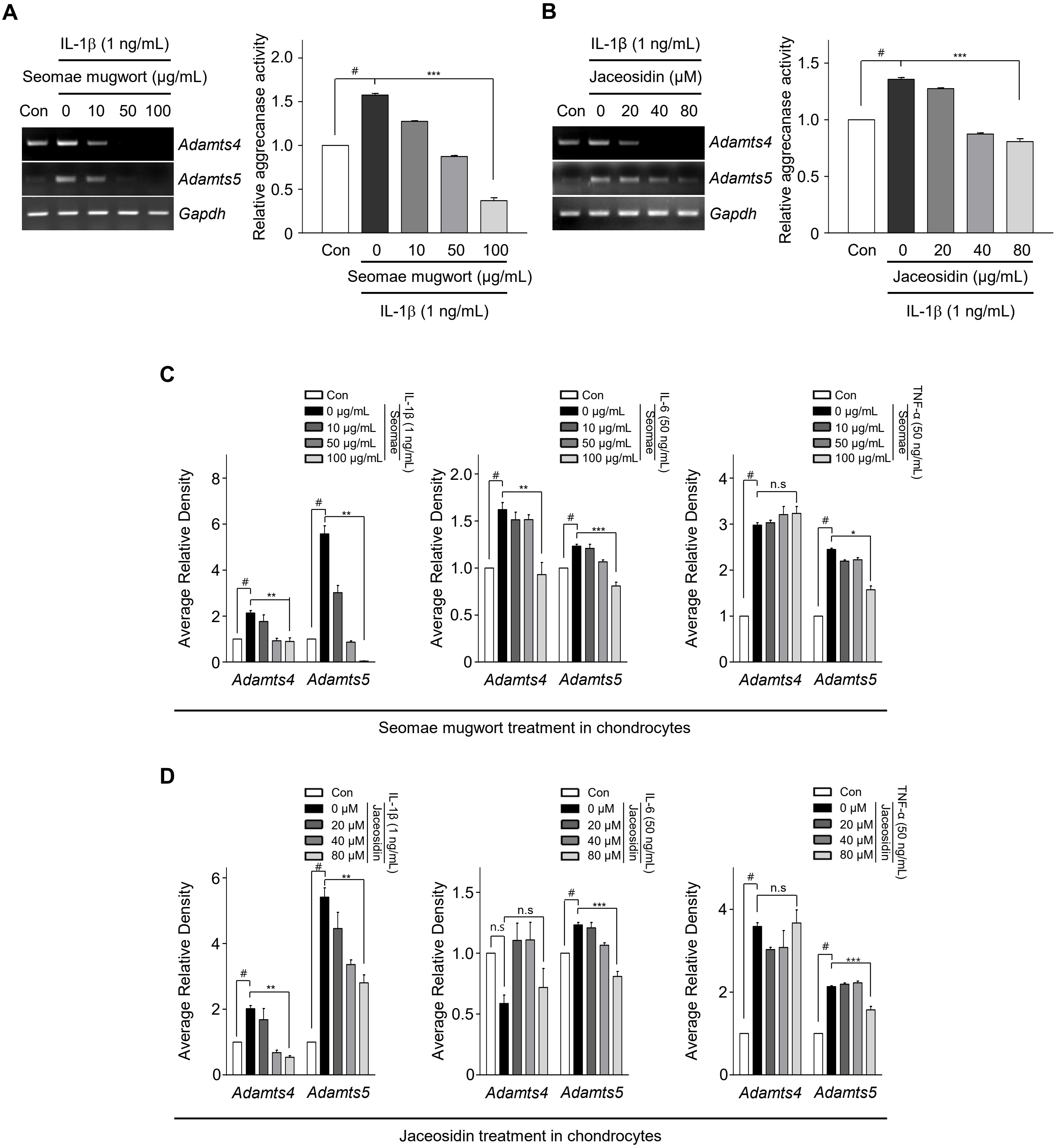
A
IL-1β (1 ng/mL)
Seomae mugwort (μg/mL)
Con 0 10 50 100
Adamts4
Adamts5
Gapdh
Relative aggrecanase activity
Seomae mugwort (μg/mL)
IL-1β (1 ng/mL)
B
IL-1β (1 ng/mL)
Jaceosidin (μM)
Con 0 20 40 80
Adamts4
Adamts5
Gapdh
Relative aggrecanase activity
Jaceosidin (μg/mL)
IL-1β (1 ng/mL)
C
Con
0 μg/mL
10 μg/mL
50 μg/mL
100 μg/mL
Seomae
IL-1β (1 ng/mL)
IL-6 (50 ng/mL)
TNF-α (50 ng/mL)
Average Relative Density
Adamts4 Adamts5
n.s
Seomae mugwort treatment in chondrocytes
D
Con
0 μM
20 μM
40 μM
80 μM
Jaceosidin
IL-1β (1 ng/mL)
IL-6 (50 ng/mL)
TNF-α (50 ng/mL)
Average Relative Density
Adamts4 Adamts5
n.s
Jaceosidin treatment in chondrocytes

Figure S3. Seomae mugwort extract and jaceosidin suppressed the IL-1β-, IL-6-, and TNF-a-induced ADAMTS4 and ADAMTS5 expression in chondrocytes. Chondrocytes were treated with IL-1β (1 ng/mL) and co-treated with different concentrations of the Seomae mugwort extract for 24 h (**A**) and jaceosidin for 12 h (**B**). ADAMTS4 and ADAMTS5 expression levels were evaluated by PCR (**A, B**; left) and aggrecanase assay (**A, B**; right) analyses. (**C, D**) Chondrocytes were treated with IL-1β (1 ng/mL), IL-6 (50 ng/mL), and TNF-α (50 ng/mL), respectively, and co-treated with Seomae mugwort extract and jaceosidin. ADAMTS4 and ADAMTS5 were detected by qRT-PCR in Seomae mugwort (**C**) and jaceosidin (**D**) treatment. Values are the mean ± SEM as analyzed by one-way ANOVA with Bonferroni's test ($n = 4$). Significant differences from the pro-inflammatory cytokine-treated group value: **$P < 0.01$, ***$P < 0.001$, #$P < 0.05$ compared to the control group. Seomae, Seomae mugwort.

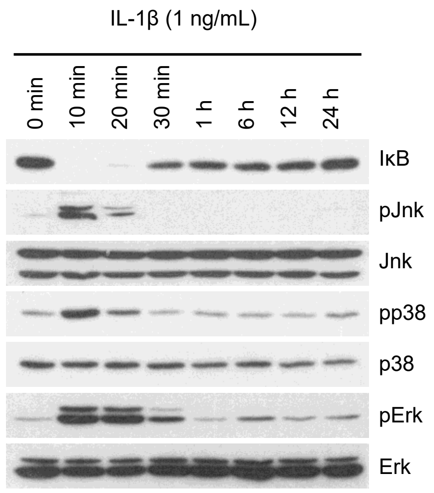

Figure S4. IL-1β induced NF-κB and MAPK signaling in mouse chondrocytes. Chondrocytes were treated with IL-1β (1 ng/mL) for different durations of time from 10 min to 24 h. The activation of each signaling pathway is shown by western blot.
